# Image-based deep learning model using DNA methylation data predicts the origin of cancer of unknown primary

**DOI:** 10.1016/j.neo.2024.101021

**Published:** 2024-06-28

**Authors:** Jinha Hwang, Yeajina Lee, Seong-Keun Yoo, Jong-Il Kim

**Affiliations:** a Department of Laboratory Medicine, Korea University Anam Hospital, Seoul, the Republic of Korea; b Department of Biomedical Sciences, Seoul National University Graduate School, Seoul, the Republic of Korea; c Genomic Medicine Institute, Medical Research Center, Seoul National University, Seoul, the Republic of Korea; d Precision Immunology Institute, Icahn School of Medicine at Mount Sinai, New York, NY 10029, USA; e Department of Oncological Sciences, Tisch Cancer Institute, Icahn School of Medicine at Mount Sinai, New York, NY 10029, USA; f Department of Artificial Intelligence and Human Health, Icahn School of Medicine at Mount Sinai, New York, NY 10029, USA; g Icahn Genomics Institute, Icahn School of Medicine at Mount Sinai, New York, NY 10029, USA

**Keywords:** Cancer unknown primary, Deep learning, DNA methylation, Molecular diagnosis

## Abstract

Cancer of unknown primary (CUP) is a rare type of metastatic cancer in which the origin of the tumor is unknown**.** Since the treatment strategy for patients with metastatic tumors depends on knowing the primary site, accurate identification of the origin site is important. Here, we developed an image-based deep-learning model that utilizes a vision transformer algorithm for predicting the origin of CUP. Using DNA methylation dataset of 8,233 primary tumors from The Cancer Genome Atlas (TCGA), we categorized 29 cancer types into 18 organ classes and extracted 2,312 differentially methylated CpG sites (DMCs) from non-squamous cancer group and 420 DMCs from squamous cell cancer group. Using these DMCs, we created organ-specific DNA methylation images and used them for model training and testing. Model performance was evaluated using 394 metastatic cancer samples from TCGA (TCGA-meta) and 995 samples (693 primary and 302 metastatic cancers) obtained from 20 independent external studies. We identified that the DNA methylation image reveals a distinct pattern based on the origin of cancer. Our model achieved an overall accuracy of 96.95 % in the TCGA-meta dataset. In the external validation datasets, our classifier achieved overall accuracies of 96.39 % and 94.37 % in primary and metastatic tumors, respectively. Especially, the overall accuracies for both primary and metastatic samples of non-squamous cell cancer were exceptionally high, with 96.79 % and 96.85 %, respectively.

## Introduction

Cancer of unknown primary (CUP) is uncommon type of metastatic cancer where the origin of the tumor is not known after detailed investigations [1]. Although recent advances in radiological and molecular assessments have led to a higher identification rate of primary tumor sites and have reduced the proportion of patients with cancer diagnosed with CUP to 1-2 %, there are still cases where the primary site of the caner is undefined [2,3]. Patients with CUP who received empirical chemotherapy were observed to have median overall survival durations ranging from 2.7 to 10.7 months [3].

The relatively poor survival observed in patients with CUP compared to patients with metastatic cancer originating from a known primary tumor suggest the importance of accurately identifying the primary tumor types for application of appropriate treatment [4]. In a meta-analysis study that evaluated the efficacy of tumor type-specific therapy in patients with CUP, the evidence is currently insufficient to recommend tumor type-specific therapy as a standard treatment approach in CUP. However, certain patients with CUP may still derive benefits from site-specific therapy [5]. Since the treatment approach for patients with metastatic tumors is largely determined by knowledge of the primary site, it is crucial to identify the primary site of the tumor to provide accurate clinical management [6,7].

Advances in machine learning algorithms [8] have led to the development of various diagnostic or prognostic methods based on medical and molecular data, which have shown more reliable and reproducible performance than conventional methods [6,9,10]. In the diagnostic work of CUP tumors, recent studies have proposed the classification model for identification of the tissue of origin for CUP based on molecular profiling or scanned hematoxylin and eosin whole-slide images (WSI) (Table 1). Tumour Origin Assessment via Deep Learning (TOAD) is a deep learning model that uses WSIs to predict the tissue of origin for CUP [11]. TOAD enables prediction of origin of CUP using routinely obtained WSI without the need for additional molecular profiling, but exhibits relatively lower accuracy compared to models that use molecular profiles. In many pan-cancer studies, each tumor type displays a distinct molecular landscape [[12], [13], [14], [15], [16]]. Based on the understanding that these molecular profiles of the primary tumor are retained in metastatic cancer, the molecular feature such as DNA mutation signatures, gene expression patterns, or DNA methylation of metastatic tumors has been utilized in several studies to predict the tissue of origin for CUP [[17], [18], [19], [20], [21], [22]]. These CUP classification models employ a variety of machine learning algorithms to identify the tissue of origin by comparing the molecular characteristics of CUP to a reference dataset of tumors with known origins. CUP-AI-Dx clearly demonstrates the applicability of image deep learning model on molecular features for CUP classification. This model showed high accuracy for identifying tissue of origin using 1D Inception convolutional neural network model and gene expression data [17].

**Table 1:** Performance of CUP classification model published previously.

**Refs.**	**Year**	**Data type**	**Method**	**Performance of external validation dataset**		
				**Accuracy**	**Validation tumor**	**# of tumor types**
[21]	2011	RT-PCR	K-nearest neighbor	83 % (187)	P + M	28
[33]	2011	Microarray	Machine learning	88.5 % (462)	P + M	15
[19]	2016	DNA methylation microarray	Random forest	94 % (534)	M	21
[32]	2020	Targeted DNA sequencing	Random forest	74.1 % (11644)	P + M	22
[22]	2020	DNA methylation microarray	deep neural network	not measured (581)	P+M	10
[17]	2020	Gene expression	1d-inception	86.96 % (23) / 72.46 % (69)	M	6 / 18
[18]	2020	Whole genome sequencing	deep neural network	88 %P / 83 %M (2120)	P + M	16
[11]	2021	Whole slide image	multitask neural network	79.9 % (682) / 61 % CUP (317)	M + CUP	17
[20]	2022	Whole genome sequencing	Random forest	58 % CUP (141)	CUP	-
Our model	2023	DNA methylation microarray	Vision transformer	96.4 %P (693) / 94.4 %M (302)	P + M	14

RT-PCR: Reverse transcription polymerase chain reaction; P: primary tumor; M: metastatic tumor; CUP: cancer unknown primary

In this study, we proposed a deep learning model based on vision transformer (ViT) [23] to predict the tissue of origin of CUP by classifying DNA methylation image patterns. We separated the non-squamous cancer and squamous cell cancer to create tumor type-specific images for each group, and then combined these two images to create a DNA methylation image for model training. This approach showed excellent classification accuracy in predicting the primary site of metastatic cancer when compared to previous published models that relied on molecular profiles.

## Materials and methods

### Data collection and preprocessing

DNA methylation data (Illumina human methylation 450k BeadChip) and clinical information of The Cancer Genome Atlas (TCGA) dataset consisting of 8,233 primary tumor samples across 31 solid tumor types were obtained from Xena platform [24]. Since the Illumine 450K array and EPIC array are frequently used to confirm genome wide analysis of DNA methylation, the CpG probes which are included in both platforms were used to further analysis. We then excluded probes with less than 80 % of samples and replaced missing values with median. The variance was calculated for each probe among the 8,233 samples and the 10,000 most variably methylated CpG probes were selected. We used Uniform Manifold Approximation and Projection (UMAP) to visualize TCGA samples in lower dimensions. Based on the results of UMAP projection, we categorized 29 cancer types to 18 tissue types (additional file 1: Table S1) and excluding two cancer types (adrenocortical cancer and uveal melanoma) with fewer than 100 cases. After excluding two cancer types, 8,074 samples were divided to 4,860 training samples (60 %), 1,600 validation samples (20 %), and 1,614 test samples (20 %).

We obtained additional 20 microarray datasets from GEO for external validation of our models. These external validation datasets consisted of 693 primary tumor and 302 metastatic tumor samples across 16 cancer types. Detailed information of GEO dataset was summarized in additional file 1: Table S2. Data preprocessing was conducted using the computing server at the Genomic Medicine Institute Research Service Center.

### Feature selection for deep learning model

We utilized the training set (n = 4,860) from TCGA to select features for the model. In UMAP analysis, since squamous cell types of cancer formed a single cluster regardless of the organ of origin, we divided the training samples into non-squamous cell cancer group and squamous cell cancer group for a more precise identification of tumor type specific differentially methylated CpGs (DMCs). For each group, we identified DMCs by calculating the median beta value of each probe in the in-class sample and comparing it to the out-of-class samples. The statistical significance assessed using Mann-Whitney U test (p<0.001).

We selected 136 DMCs for each of the 17 non-squamous cell cancer subtypes and 90 DMCs for each of the 5 squamous cell cancer subtypes. As a result, we used 2,312 DMCs from the non-squamous group and 450 DMCs from the squamous group to generate the image for the deep learning model.

### Transformation of DNA methylation data to images and build the vision transformer model

We used the Image Generator for Tabular Data (IGTD) tool [25] to generate images from the tabular data of DMCs using the Euclidean distance method with 5,000 iterations. The 2,312 DMCs from the non-squamous group were converted into images with a size of 68×34, while the 450 DMCs from the squamous group were converted into 30×15 size images. The DNA methylation image of the squamous group was resized to 68×34 and merged with the non-squamous part image. To ensure compatibility with image data standards, the values in the image data were scaled to a range of 0 to 255, and since the image data is 3-channel, the 68×68 DNA methylation data array was multiplied by 255 and repeated three times to create a grayscale image in a 3-channel format (68×68×3).

We employed the ViT model to predict the class of images from DNA methylation data. The model architecture was based on the basic ViT using Tensorflow and vit-keras python package. The input image is divided into the 289 (17×17) image patches, which were flattened into a vector. Position embedding vectors were added to the patch embedding vectors, and the resulting vectors were passed through multiple transformer blocks that utilized multi-headed attention layers. We used 8 transformer blocks with the four attention heads, and the projection dimension was set to 256. We used two fully connected layers with 512 and 256 neurons, and we utilized the GELU as activation function in the transformer block. The final output of the transformer block was flattened and served as the input vector for two fully connected layers with 1024 and 512 neurons. The model was trained using a batch size of 100, epoch of 14, learning rate of 0.0001, and the AdamW optimizer. We used a hold-out validation method for training this model.

Direct visualization of the attention in the model is another notable feature of the ViT model. Following a similar approach described in a self-supervised learning method for ViT, we used the attention weights of multi-head in the final layer of the Transformer encoder to visualize the attention patterns.

#### Model performance evaluation

Overall accuracy, precision, recall, and f-1 score were calculated to evaluate the performance. The performance metrics were computed as follows:

$$Accuracy=\;\frac{True\;positive+True\;negative}{True\;positive+False\;negative+False\;positive+True\;negative}$$

$$Precision=\;\frac{True\;positive}{True\;positive+False\;positive}$$

$$Recall=\;\frac{True\;positive}{True\;positive+False\;negative}$$

$$f1\;score=2\times \frac{Precision\times Recall}{Precision+Recall}$$

## Results

### Classification of tumor origin based on deep learning model

**Fig. 1:**
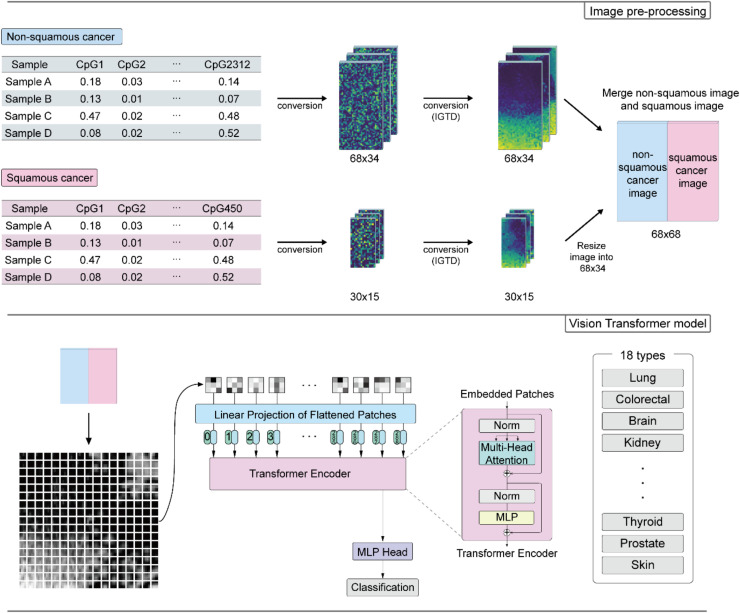
**CUP-classifier model workflow.** The beta values of non-squamous cancer-specific DMCs and squamous cancer-specific DMCs were converted into 68×34 and 30×15 array formats, respectively, and were followed by conversion to IGTD image. The squamous part image was resized to 68×34 and merged with non-squamous part image. Merged images were divided into 17×17 image patches and these patches were used to train the ViT model for classification of CUP.

First, we used DNA methylation microarray data from the TCGA, consisting of 8,233 primary tumor samples, to investigate the global DNA methylation patterns across different types of cancer. Upon visualizing the data using UMAP, we observed distinct groupings of samples based on their respective cancer types. Also, we found that cancer clusters exhibiting the same organ origin or histological feature tended to be located in close proximity (additional file 2: Fig. S1). For the most cancers exhibited clustering patterns based on organ types, such as colorectal (colon adenocarcinoma and rectum adenocarcinoma), oesophagogastric (esophageal carcinoma and stomach adenocarcinoma), kidney (kidney papillary cell carcinoma, kidney clear cell carcinoma, and kidney chromophobe), hepatobiliary (liver hepatocellular carcinoma and cholangiocarcinoma), brain (brain lower grade glioma and glioblastoma multiforme), soft tissue (Sarcoma and mesothelioma) and gynecologic cancer (uterine corpus endometrioid carcinoma, uterine carcinosarcoma, ovarian serous cystadenocarcinoma, and cervical & endocervical cancer). In contrast, some other cancers were clustered based on histological features, such as squamous cell cancers (head & neck squamous cell carcinoma, esophageal carcinoma, lung squamous cell carcinoma, cervical & endocervical cancer, and bladder urothelial carcinoma). Based on these results, we excluded two cancer types with small cohorts (79 adrenocortical cancers and 80 uveal melanomas) and re-categorized 29 cancer types into 18 common origin classes according to their primary organ or histological characteristics. Subsequently, we built a deep learning model for classification of tissue origin of cancer (additional file 1: Table S1) (Fig. 1).

**Fig. 2:**
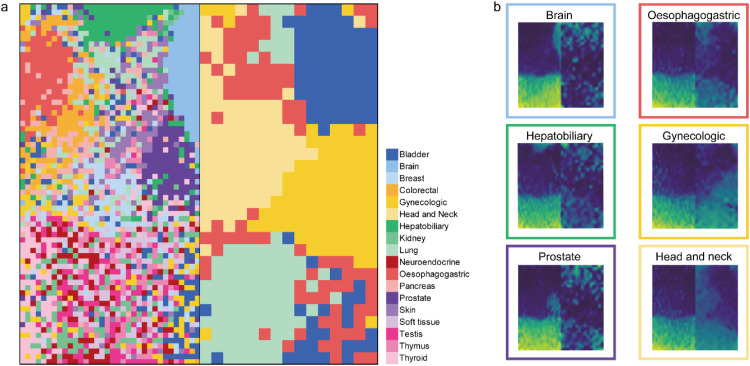
**Differentially methylated CpG (DMC) map and DNA methylation image.** (a) Detailed pixel location of DMCs in the DNA methylation image, colored by tissue type. (b) Example of DNA methylation image of six tissue types and outline of the image was colored by tissue type.

We divided a total of 8,074 tumor samples into training (60 %), validation (20 %), and test datasets (20 %), comprising 4,860, 1,600, and 1,614 samples, respectively. Using the training dataset, we independently identified class-specific DMCs for the non-squamous group and squamous group. To enhance the performance of our model, we transformed the tabular format of beta values into an image format using the IGTD tool. In this transformation, we assigned similar features to neighboring pixels and dissimilar features to pixels that are far apart, taking into consideration the similarity of DNA methylation value among the probes. We found that the pixel location of certain class-specific DMCs, such as brain, oesophagogastric, hepatobiliary, and prostate, were clustered together within the same tissue type (Fig. 2). To generate a unique image representing the characteristic of the both class-specific DMCs from non-squamous group and squamous group, we combined the DNA methylation image of the squamous group with the non-squamous part image and used the combined image as an input for the model. Each tumor sample exhibited a unique image pattern in the DNA methylation data. When we averaged the DNA methylation images for each class, we observed distinct patterns that were specific to the origin of cancer (Fig. 2 and additional file 2: Fig. S2). The image patterns of randomly selected individual sample differed slightly from each other, however, the tissue-specific patterns were confirmed in most samples (additional file 2: Fig. S3).

The DNA methylation images, generated using the IGTD tool, were used to train the ViT model. After training the classifier, we further examined the relative significance of the regions analyzed by the model in terms of human interpretability.

### Evaluation of model performance and model interpretability

**Fig. 3:**
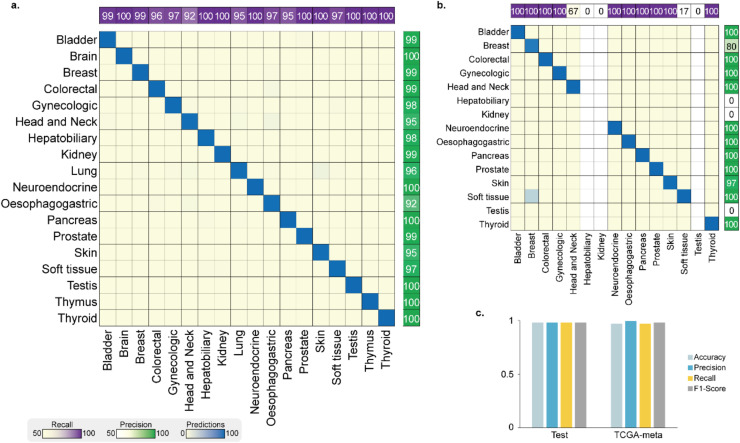
**Performance for the prediction of the tumor origin on the TCGA dataset.** (a) The classifier model confusion matrix for TCGA test dataset and (b) TCGA-meta datasets. Rows and columns of the matrix represent the predicted classes by the model and the true classes of the tumor, respectively. The number of samples and recall are plotted upper of the matrix and precision is plotted next to the confusion matrix. (c) Overall accuracy and the weighted average of precision, recall, and F1-score of the classifier for the test dataset (left) and TCGA-meta dataset (right).

We evaluate the performance of our model using a test dataset of 1,614 primary tumors and 394 TCGA metastatic tumors (TCGA-meta) that were not used in the model training. The performance metrics for test and TCGA-meta dataset are shown in Fig. 3a and Fig. 3b, respectively. The model demonstrated an overall accuracy of 97.96 % for the test dataset, and 96.95 % for the TCGA-meta dataset, highlighting its strong performance. In addition, the weighted average of precision, recall, and F1-score exceeded 0.97 for both the test dataset and TCGA-meta dataset (Fig. 3c).

The predictive performance of TCGA-meta dataset was comparable to that of the test datasets. These findings suggest that metastatic samples maintain the molecular profile of the primary tumor, enabling the model to make accurate predictions regarding the primary site of the tumor.

For interpretability of the CUP classifier, attention map visualizations were generated for self-attention in the transformer encoder. additional file 2: Fig. S4 shows the average of attention maps for each class. Our model revealed that the attention of the model mainly focuses on regions with unmethylated probes.

### Application of classifier to external datasets

To expand the utilization of our model in a various clinical setting, we analyzed the performance of our model on an external validation data set consisting of 995 samples (693 primary and 302 meta samples) across 14 primary cancer types from 20 independent studies (additional file 1: Table S2). Without data normalization or model tuning, our classifier model achieved overall accuracy at 96.39 % in the 693 primary tumors and 94.37 % in the 302 metastatic tumors. Confusion matrix and performance metrics for each class are shown in additional file 2: Fig. S5.

**Fig. 4:**
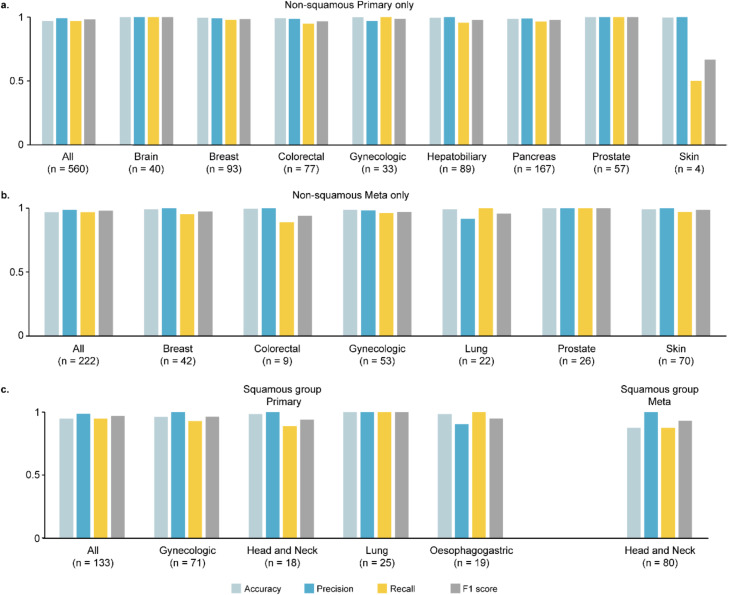
**Performance for the external validation datasets.** Metrics including per-class accuracy, precision, recall, F1-score are calculated for the (a) primary tumors, (b) metastatic tumors in non-squamous group, and (c) primary and metastatic tumors in squamous group. Overall accuracy, weighted average of precision, recall, F1-score for each dataset are plotted at the front of the plot.

Next, we divided the external dataset into a non-squamous cancer group and a squamous cell cancer group to assess the performance of each group independently. In the non-squamous cancer group, there were 560 primary cancer samples from 8 cancer types and 222 metastatic cancer samples from 6 cancer types. The overall accuracy for both primary and metastatic samples was remarkably high, with 96.79 % and 96.85 % accuracy, respectively. The squamous cell cancer group consisted of 133 primary cancer samples from 4 squamous cell cancer types and 80 metastatic head and neck cancer samples. The overall accuracy for primary samples was 94.74 %, while for metastatic samples, it was 87.5 % (Fig. 4).

**Fig. 5:**
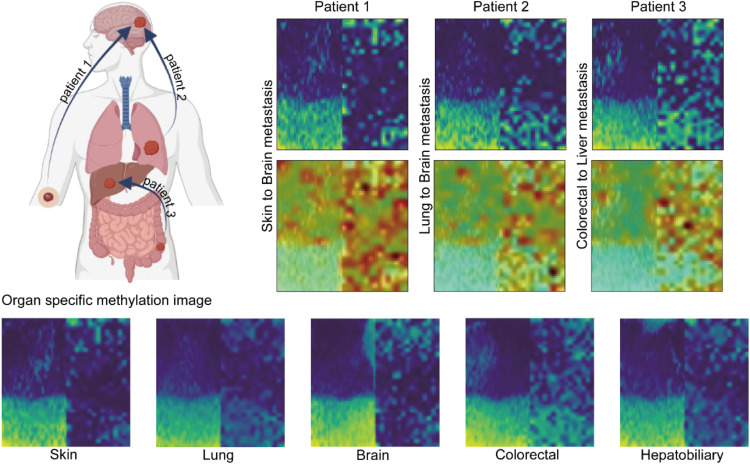
**DNA methylation image of metastatic cancer.** Two brain metastases were selected for example: one from melanoma (patient 1) and another from lung adenocarcinoma (patient 2). Additionally, one liver metastasis from colorectal adenocarcinoma (patient 3) was included. Original DNA methylation image of five tissues (lung, skin, brain, colorectal, and hepatobiliary) were located in bottom of the figure.

We identified that DNA methylation images of brain metastasis samples originated from melanoma and lung showed that each sample resembled its primary site, skin and lung pattern, rather than the brain. Similarly, in the case of liver metastasis originating from colorectal cancer, the DNA methylation pattern corresponded with the colorectal pattern rather than liver image. These results indicated that the DNA methylation heatmap pattern of the metastatic samples retained the primary organ pattern rather than reflecting the metastasized organ (Fig. 5).

## Discussion

Deep learning technology has undergone significant advancements and has been extensively explored in the analysis of biological and clinical data. Notably, the field of image deep learning has witnessed substantial progress in the diagnosis of diseases and identification of lesions using medical imaging data, such as X-rays, CTs or pathology slides [26,27]. Furthermore, deep learning has also been applied to various biomedical fields utilizing omics data, including variant calling, annotation, and prediction of pathogenic variants [[28], [29], [30], [31]].

Multiple methods have been developed to classify the CUP, utilizing diverse types of data, such as WSI, DNA methylation microarray, and RNA or DNA sequencing data. Marker gene analysis or traditional machine learning techniques such as random forest, regression, and support vector machine were used to train the model for classification [[18], [19], [20], [21],^32^,^33^]. In recent studies, deep learning methods such as deep neural networks and 1d-inception algorithm have been applied to identify the origin of unknown primary cancer [11,17,22].

In this study, we proposed a deep learning algorithm designed for the classification of CUP based on the analysis of organ-specific image patterns derived from DNA methylation microarray data. DNA methylation is generally less sensitive to batch or platform variations compared to RNA expression data, requiring less extensive data normalization. We trained the model using DNA methylation images and confirmed that our model exhibited excellent performance on both the test dataset and the TCGA-meta dataset. To validate the effectiveness of our model, we performed validation using data from 20 independent studies without additional model tuning and data normalization, ensuring that its performance is robust and can be applied effectively to various clinical settings.

Our model demonstrated high accuracy compared to what has been reported in other studies in the external validation dataset. Specifically, it achieved an accuracy of 96.39 % and 94.37 % for primary cancer and metastatic cancer dataset, respectively.

The models proposed in previous studies have been evaluated with an external dataset primarily consisting of non-squamous cancer datasets. For example, EPICUP which also predicts CUP based on DNA methylation data showed 94 % accuracy on the external dataset; however, it only included 11 squamous cancers (5 cervical squamous carcinoma and 6 Head & Neck squamous cell carcinoma) out of 534 metastatic cancer datasets. In case of study conducted by Zheng et al, the performance of model was evaluated with 581 independent cancer samples, but only 6 squamous cancer samples were included in the dataset.

When our model was specifically applied to non-squamous cancer samples, it demonstrated an exceptional performance with 97 % accuracy in both of 560 primary tumor samples and 222 metastatic tumor samples. Although the number of tested metastatic cancer were smaller than EPICUP, our model has given the best accuracy than any other previous model before. The squamous cancer group showed 95 % accuracy for primary tumor samples and 88 % accuracy for metastasis tumor samples, which was slightly lower compare to non-squamous group.

While our model demonstrated strong performance in independent datasets consisting of metastatic cancer, we have several limitations in testing our model. First, we evaluated our model with a dataset composed of many types of cancer, but we were unable to test several types of cancer and performance tests on actual CUP samples are insufficient. Although we accurately predicted four CUP samples from GSE108576, a large dataset of CUP is needed for more accurate performance evaluation. Second, our classifier showed lower performance in the squamous cell cancer group because of the lack of training data.

## Conclusions

In summary, we have constructed image-based deep learning models for predicting the origin of CUP utilizing DNA methylation data. Owing to the very nature of DNA methylation data, our model showed great performance in prediction regardless of non-squamous cell cancers or squamous cell cancers without the need for minimal data normalization. We needed more DNA methylation data of patient with CUP or squamous cell cancer for further validation. However, our model has potential for improving the efficiency and accuracy of diagnosing cases where the primary cancer site is unknown.

## CRediT authorship contribution statement

**Jinha Hwang:** Conceptualization, Data curation, Formal analysis, Investigation, Methodology, Project administration, Resources, Software, Supervision, Validation, Visualization, Writing – original draft. **Yeajina Lee:** Conceptualization, Data curation, Formal analysis, Investigation, Methodology, Project administration, Resources, Software, Validation, Visualization, Writing – original draft. **Seong-Keun Yoo:** Conceptualization, Funding acquisition, Project administration, Writing – review & editing, Supervision. **Jong-Il Kim:** Conceptualization, Project administration, Supervision, Writing – review & editing.

## Declaration of competing interest

The authors declare that they have no known competing financial interests or personal relationships that could have appeared to influence the work reported in this paper.

## Appendix Supplementary materials

Image, application 1

Image, application 2
