# Supplementary material for: Image-based deep learning model using DNA methylation data predicts the origin of cancer of unknown primary
Source: Neoplasia. 2024 Jun 28;55:101021. doi: 10.1016/j.neo.2024.101021 (PMC11261876; doi:10.1016/j.neo.2024.101021)
Supplement: Supplementary file 2 [file mmc2.docx]

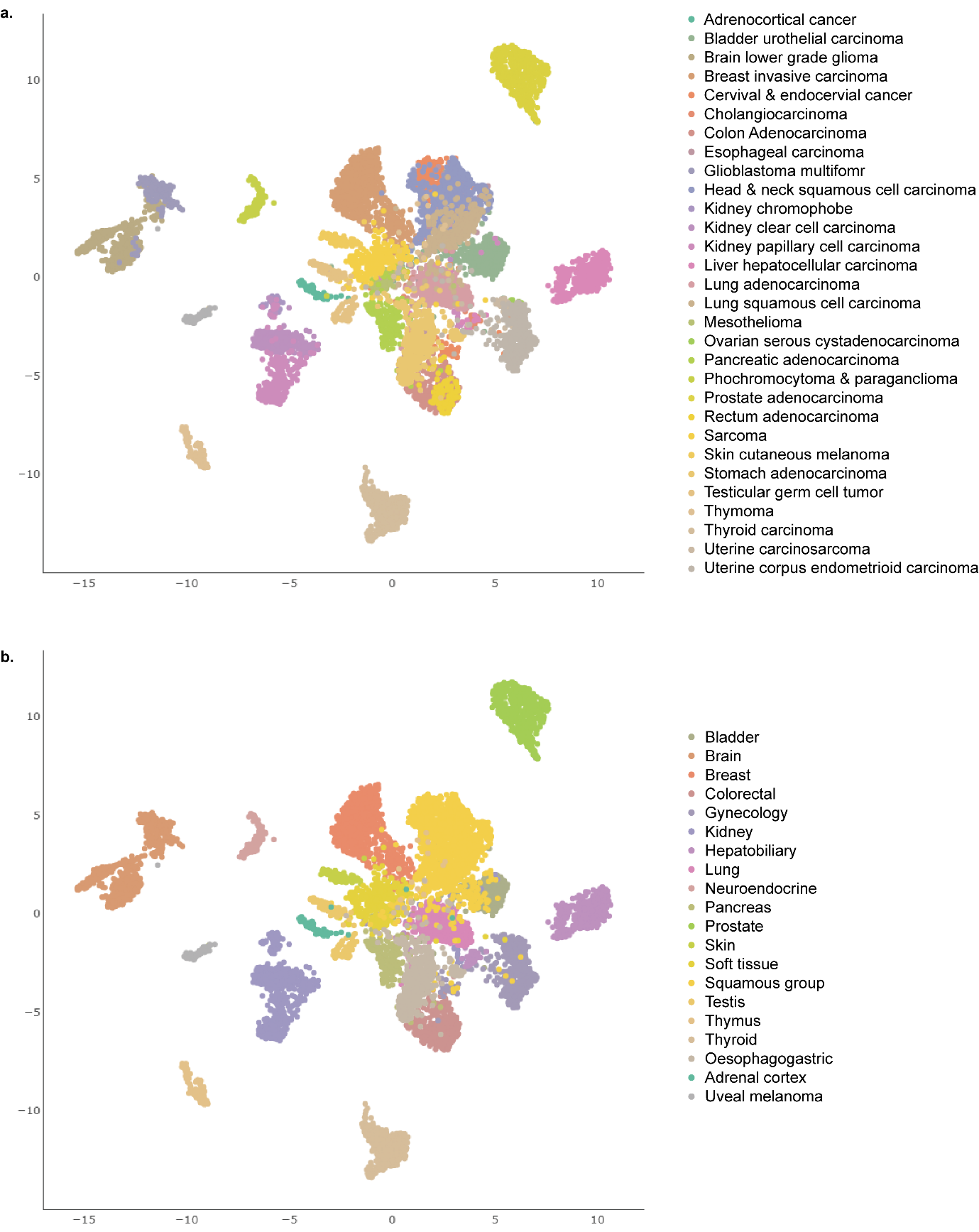


**Figure S1.** DNA methylation profile of pan-cancer TCGA. UMAP plots of the TCGA cancer samples based on DNA methylation level of most variable 10000 CpG probes, colored by cancer type (a), and re-categorized 18 tissue type (b).


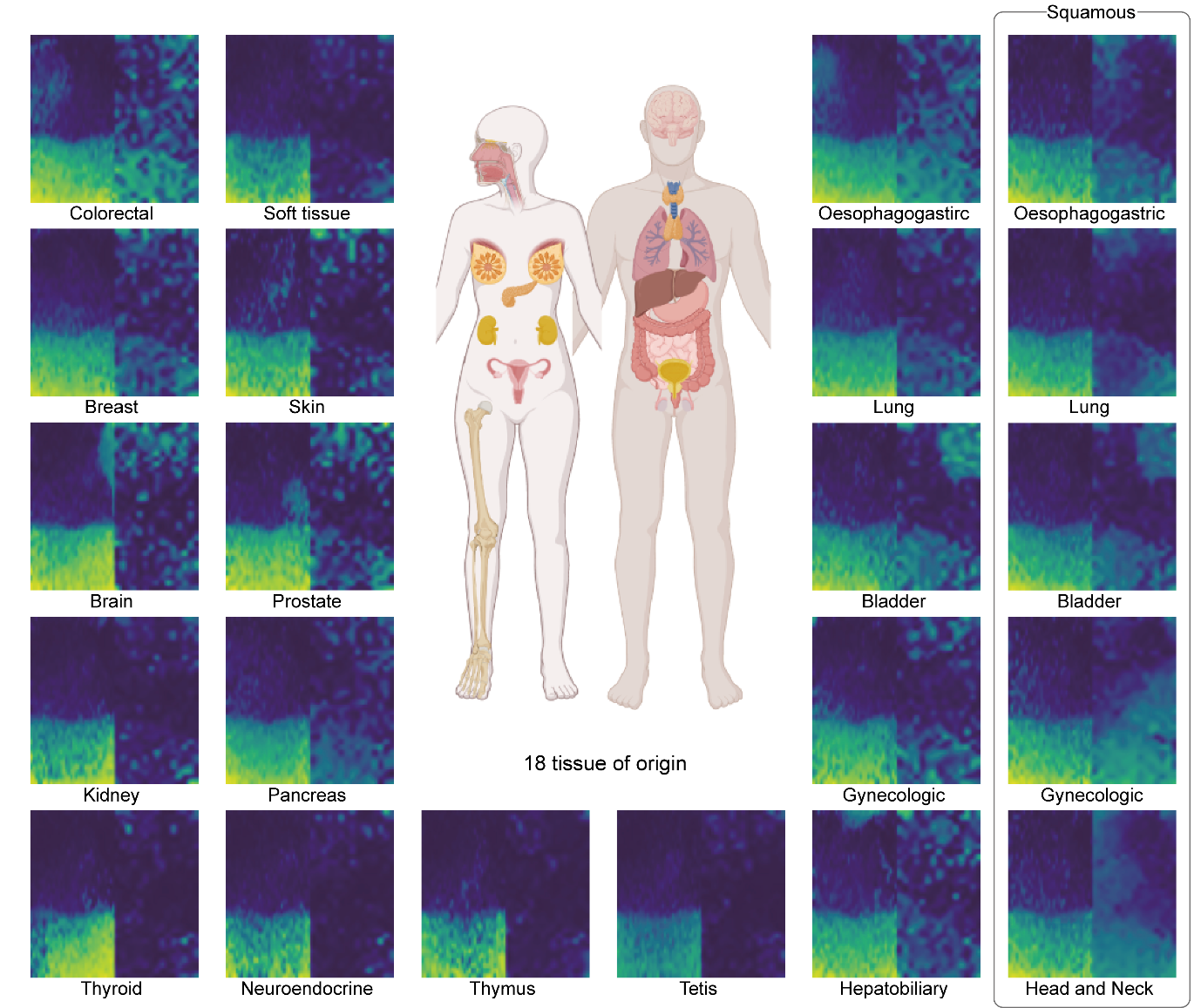


**Figure S2.** Average of DNA methylation image for 18 tissue of origin.

**
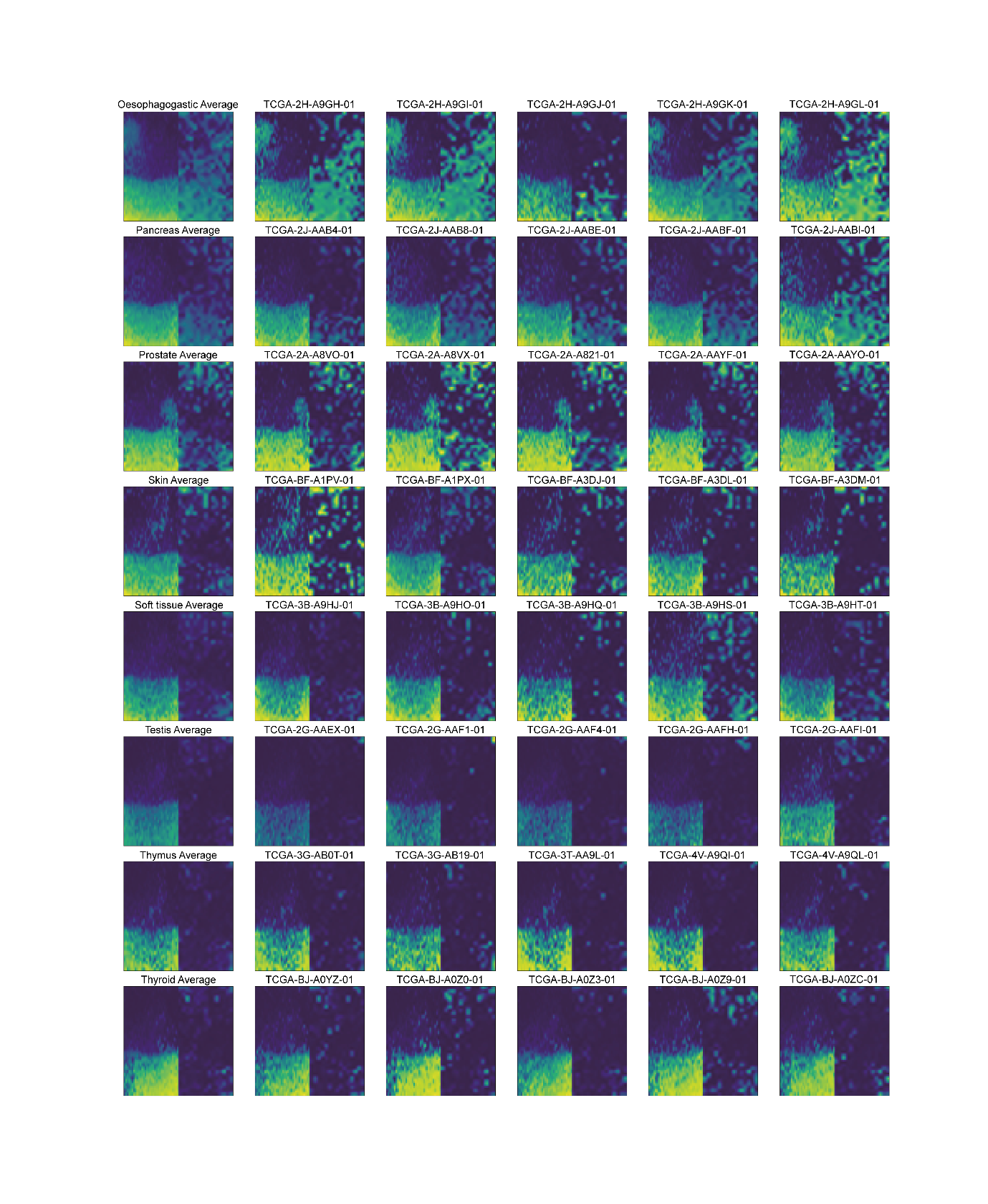
**

**Figure S3a.** DNA methylation image of individual sample. Example image of randomly selected 5 samples for each tissue of origin from non-squamous group**
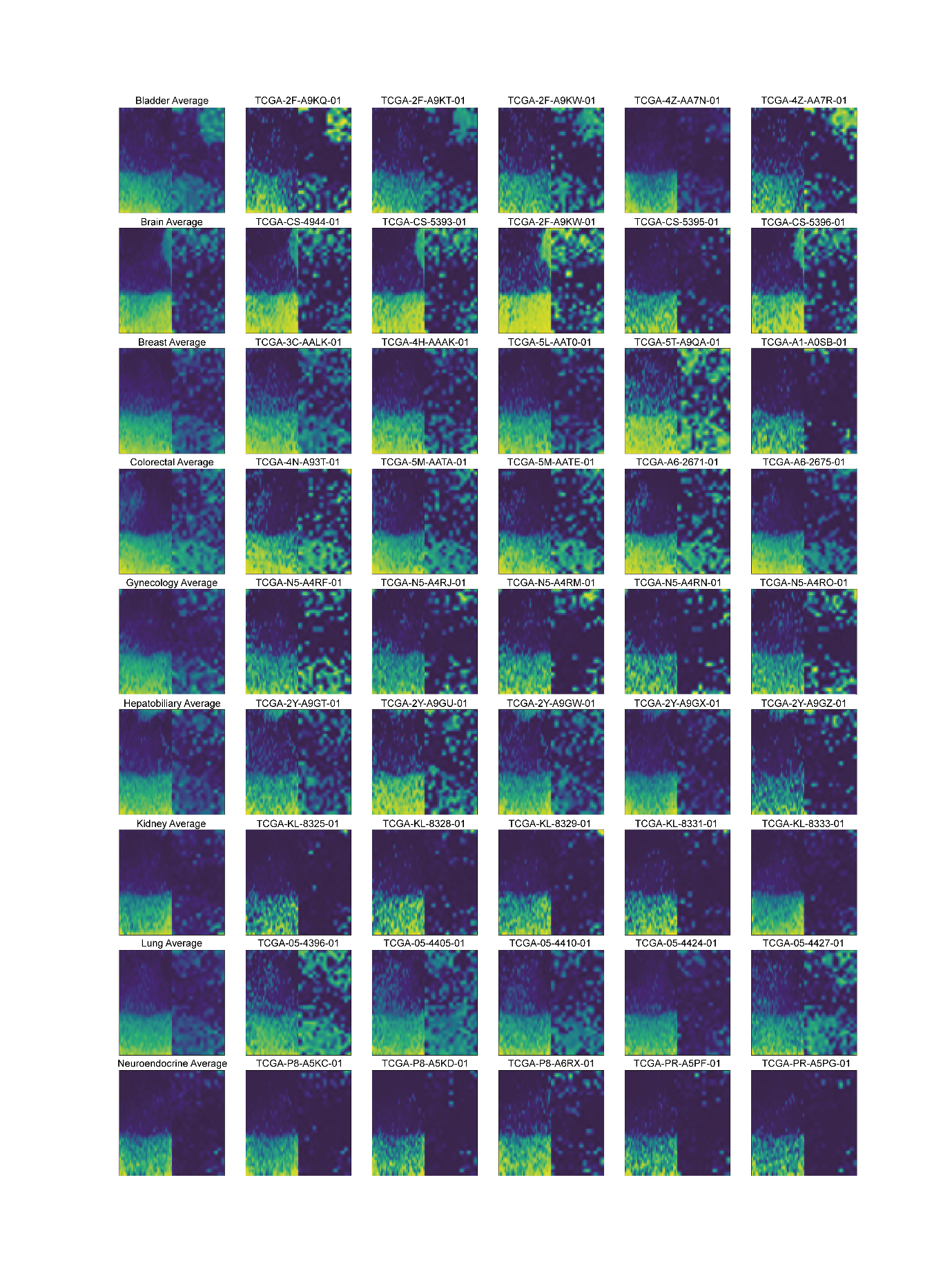
**

**Figure S3a continued.** DNA methylation image of individual sample. Example image of randomly selected 5 samples for each tissue of origin from non-squamous group


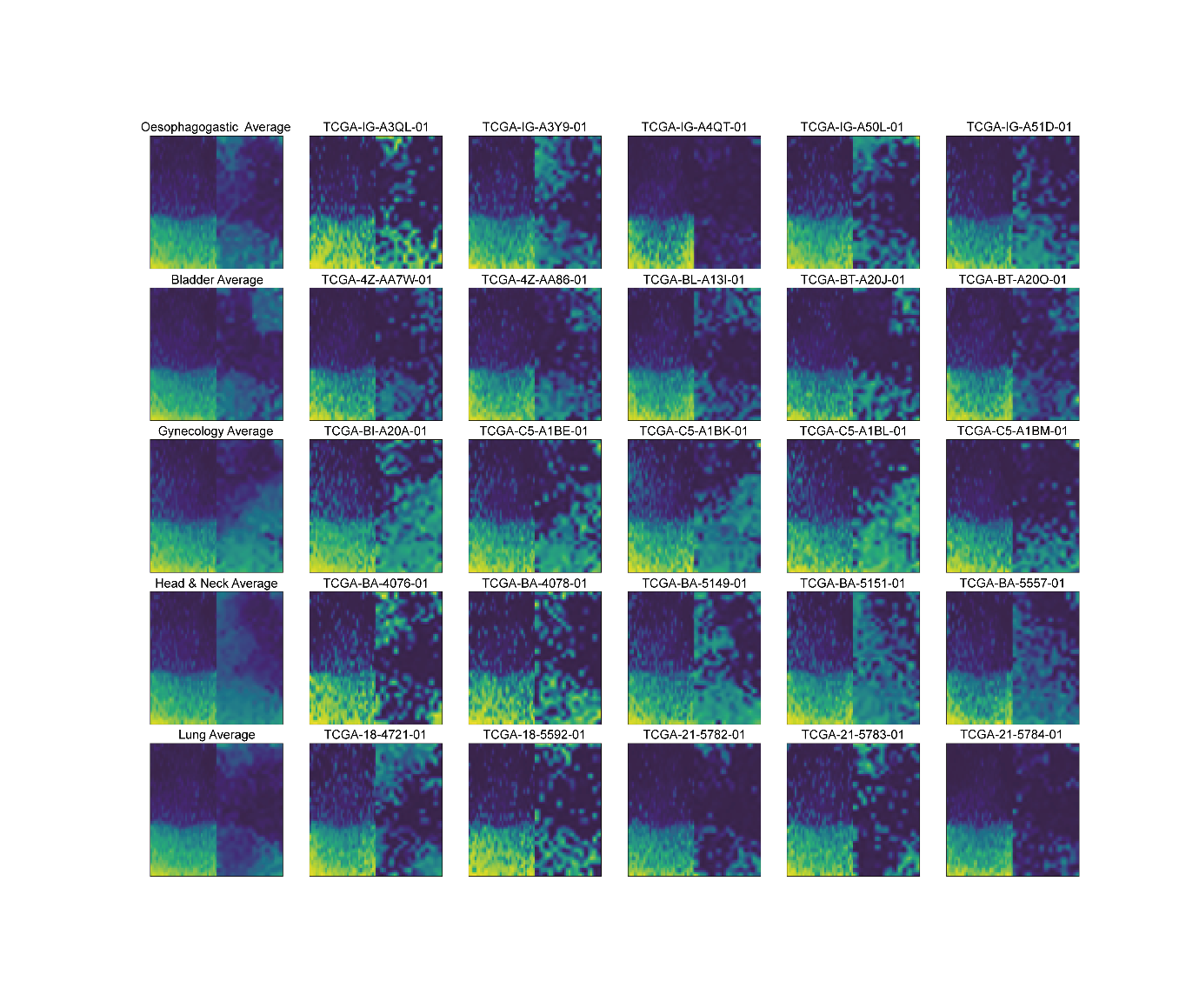


**Figure S3b.** DNA methylation image of individual sample. Example image of randomly selected 5 samples for each tissue of origin from squamous group.

**
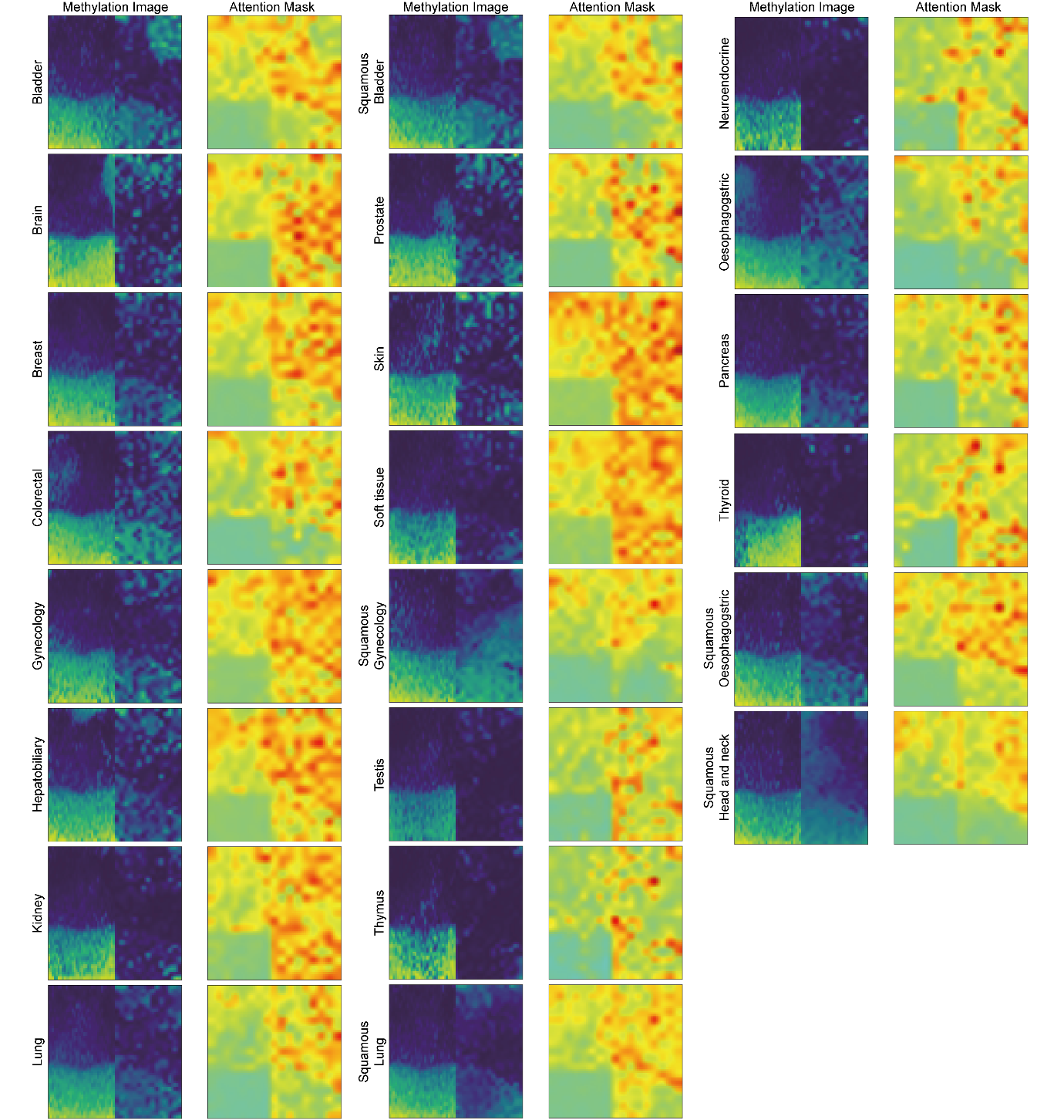
Figure S4.** Model interpretability. The attention score for each image region are used to interpret the results of the classifier model. Average of DNA methylation image was located in left and attention map was located in right for each tissue type.


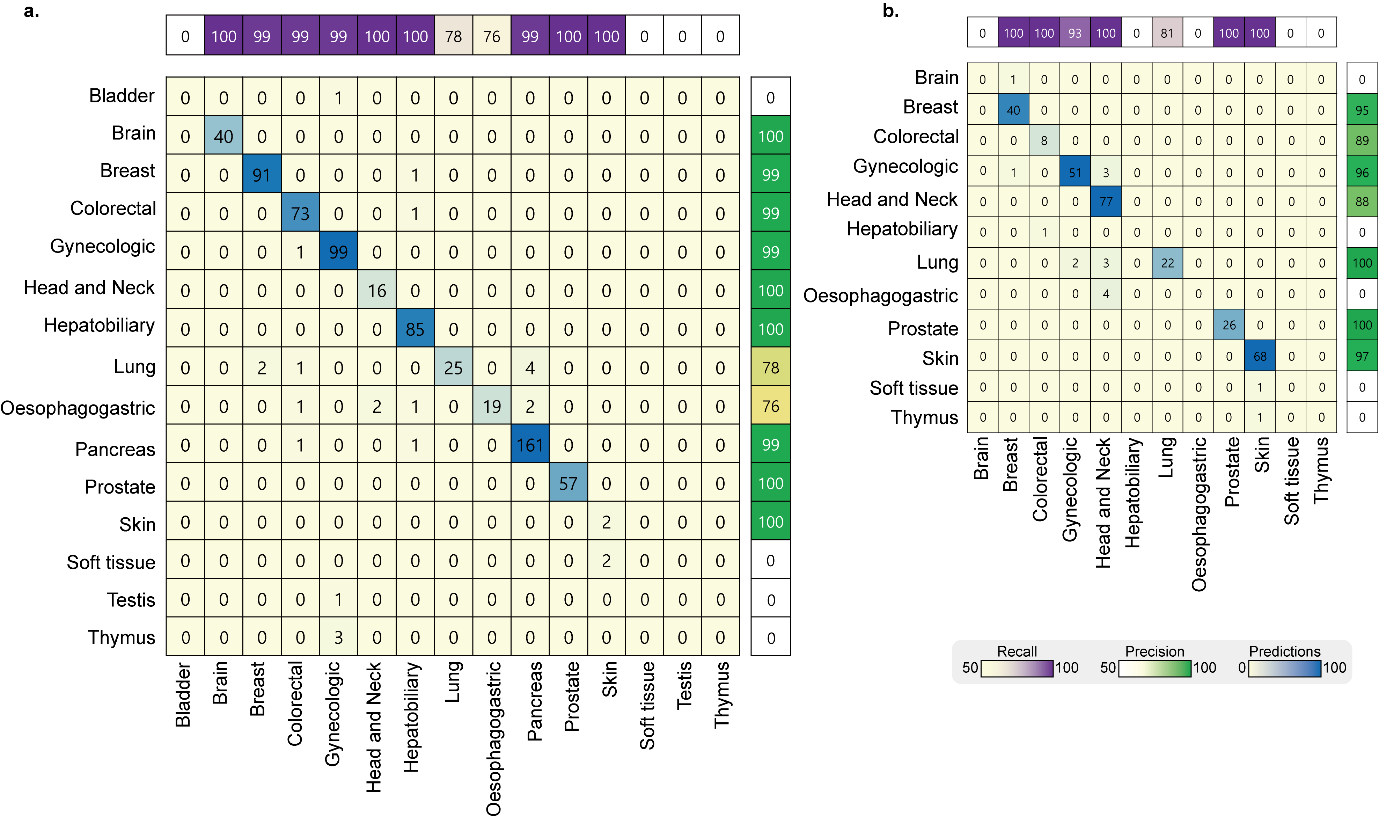


**Figure S5.** Confusion matrix of (a) primary cancer and (b) metastatic cancer dataset of external validation set. Rows and columns of the matrix represent the predicted classes by the model and the true classes of the tumor, respectively.

**Additional File 1: Table S1.** The list of original cancer types and re-categorized tissue class of TCGA DNA methylation dataset.

**Additional File 1: Table S2.** List of external data set.

**Additional File 2:** **Figure S1.** DNA methylation profile of pan-cancer TCGA. UMAP plots of the TCGA cancer samples based on DNA methylation level of most variable 10000 CpG probes, colored by cancer type (a), and re-categorized 18 tissue type (b).

**Additional File 2:** **Figure S2.** Average of DNA methylation image for 18 tissue of origin.

**Additional File 2: Figure S3.** DNA methylation image of individual sample. Example image of randomly selected 5 samples for each tissue of origin from (a) non-squamous group, and (b) squamous group.

**Additional File 2: Figure S4.** Model interpretability. The attention score for each image region are used to interpret the results of the classifier model. Average of DNA methylation image was located in left and attention map was located in right for each tissue type.

**Additional File 2: Figure S5.** Confusion matrix of (a) primary cancer and (b) metastatic cancer dataset of external validation set. Rows and columns of the matrix represent the predicted classes by the model and the true classes of the tumor, respectively.
